# Identifying Longitudinal Compliance Patterns and Determinants in a Multifaceted Childhood Obesity Intervention Using Group-Based Trajectory Modeling

**DOI:** 10.3390/nu17101701

**Published:** 2025-05-16

**Authors:** Shiyu Yan, Wenhao Li, Miaobing Zheng, Jinlang Lyu, Shuang Zhou, Hui Wang, Yan Li, Haijun Wang

**Affiliations:** 1 Department of Maternal and Child Health, School of Public Health, Peking University, National Health Commission Key Laboratory of Reproductive Health, Beijing 100191, China; yan_sy@bjmu.edu.cn (S.Y.); jinlanglyu@bjmu.edu.cn (J.L.); zhoushuang0601@bjmu.edu.cn (S.Z.); huiwang@bjmu.edu.cn (H.W.); 2 Peking University Health Science Center-Weifang Joint Research Center for Maternal and Child Health, Beijing 100191, China; 3 Institute of Child Health Care, Jinan Children’s Hospital, Jinan 250022, China; claudialwh@163.com; 4 Institute for Physical Activity and Nutrition, Faculty of Health, Deakin University, Geelong 3217, Australia; j.zheng@deakin.edu.au; 5 Department of Pediatrics, Shandong Maternal and Child Health Hospital, Jinan 250000, China

**Keywords:** obesity, intervention, compliance, trajectory, influencing factors

## Abstract

**Background/Objectives**: Identifying the factors influencing compliance is essential to improve the effectiveness of interventions. However, no study has examined factors that influence the longitudinal patterns of obesity intervention compliance. We aim to identify the longitudinal trajectories of parental and child compliance using group-based trajectory modeling (GBTM) and assess the influencing factors. **Methods**: The Diet, ExerCIse, and CarDiovascular hEalth Children (DECIDE-Children) was a 9-month app-assisted obesity prevention intervention targeted 8–10-year-old children. Altogether, 684 child–parent pairs from the intervention group were included. Parents were required to use the mobile app to learn health knowledge, monitor children’s diet and exercise behaviors, manage children’s weight, and received the assessment results. Parental compliance was assessed as the monthly usage times and duration of the mobile app. For child compliance, we used data recorded by parents in the “behavior monitoring” module. We employed group-based trajectory modeling (GBTM) to identify distinct trajectories of parental and child compliance and examined their associations with childhood obesity outcomes. Univariate and multivariate logistic regressions were performed to identify the influencing factors associated with the identified compliance groups. **Results**: Distinct trajectory groups of parental and child compliance were identified. The compliance trajectories of parents and children are related to the extent of changes in the child’s obesity-related outcomes (waist circumference, waist-to-hip ratio, and body fat percentage. *p* < 0.05). A majority of parents were classified into the “relatively low compliance” group. Parents in this group was associated with having a daughter (OR: 1.95, 95% CI: 1.17, 3.31) and the father having a higher education level (OR: 1.65, 95% CI: 1.05, 2.60). For children, 20.2% were assigned to the “decreasing compliance” group. Children in this group were more likely to have a younger mother (OR: 1.05, 95% CI: 1.01, 1.10) and parents with poorer compliance (OR: 2.36, 95% CI: 1.16, 5.47). **Conclusions**: Both student and parental compliance were shown to influence the effectiveness of childhood obesity interventions, highlighting the need to prioritize the assessment and promotion of compliance in such interventions. Child sex, paternal educational level, and maternal age were identified as significant factors associated with compliance, while the level of family involvement was found to play a pivotal role in fostering healthy behaviors in children. These findings suggest that future intervention strategies should place greater emphasis on engaging families and providing targeted supervision and support for populations at risk of lower compliance in order to enhance intervention outcomes.

## 1. Introduction

The prevalence of overweight and obesity is rapidly increasing worldwide. The World Health Organization reported that the global prevalence of overweight and obesity in childhood and adolescents (aged 5–19 years old) has risen from 4% in 1975 to over 20% in 2022 [1]. In China, from 1985 to 2019, the obesity rate increased from 0.1% to 9.6% among primary and middle school students [2]. Without intervention, the overweight and obesity rate of children will reach 28.0% by 2030 [3]. Childhood obesity is associated with a range of short- and long-term adverse consequences, including cardiovascular disease, type 2 diabetes mellitus, and several malignancies [4]. Thus, it is important to strengthen obesity prevention strategies to reverse the alarming obesity epidemic and its associated adverse consequences.

Lifestyle intervention is one of the most widely used interventions for the prevention and management of childhood obesity. Although there is moderate evidence to support the effectiveness of school-based interventions for obesity prevention, the reported intervention effects remain too small to be clinically meaningful [5]. One of the key factors influencing intervention effects is poor compliance. Compliance is defined as the extent to which a person’s behavior corresponds to the allocated intervention as intended [6]. Premature withdrawal of subjects or deviation from the intended intervention would affect the effect of the intervention and, in turn, the interpretation of the intervention effectiveness [6,7]. A better understanding of the factors influencing compliance in obesity intervention studies is essential for identifying individuals at risk of low adherence and for informing strategies to enhance participant engagement. However, limited studies examined compliance in childhood obesity intervention studies, with only 37% of compliance being reported in high-quality interventions [8]. In studies where compliance data were gathered, most used data to evaluate the acceptability and feasibility of the study protocol [9,10] or to examine how the degree of compliance influenced the target outcomes and intervention effects [11,12]. Several studies have considered factors that influence compliance such as attrition or mean number of in-person clinical visits [13,14]. However, these studies failed to capture individual changes in compliance throughout the intervention. To the best of our knowledge, no study to date assessed the longitudinal changes in compliance in childhood obesity intervention. In recent years, mobile health (mHealth) platforms have gained increasing popularity as a means of delivering childhood obesity interventions [15], while also providing researchers with more precise and longitudinal compliance data.

This study aimed to identify distinct longitudinal patterns of parental and child compliance in the 9-month childhood obesity intervention (Diet, ExerCIse, and CarDiovascular hEalth Children, DECIDE-Children) using mobile app-based data and to explore the key factors influencing compliance trajectories throughout the intervention period.

## 2. Materials and Methods

### 2.1. Study Design and Participants

The DECIDE-Children intervention was a cluster randomized controlled trial conducted in 3 socioeconomically distinct Chinese areas: Beijing, Changzhi of Shanxi Province, and Urumqi of Xinjiang Province. Details of the trial protocol have been published [16]. The study enrolled Grade 4 students (8–10 years old) from 24 schools, who were randomized to the intervention group (12 schools) or control group (12 schools) stratified by district within each region. From one to two classes were recruited from each school. Informed consent was obtained from the parents of all participating students, and parental confirmation was required to ensure that their children met the health criteria for study participation. Exclusion criteria included a history of heart disease, hypertension, pathological obesity, abnormal physical development or deformities, inability to participate in physical activities, and unexplained significant weight loss within the past three months. The intervention was based on a socio-ecological model, which was conducted in schools with family involvement. The intervention duration was 9 months (from September 2018 to June 2019). A mobile app (‘Eat Wisely, Move Happily’) (version 1.0) was used to better connect schools and families. Ethical approval was granted by the Peking University Institutional Review Board (IRB00001052-18021). For the current analyses, only children and parents in the intervention group who completely participated in the study were included. A total of 12 schools and 684 pairs of student–parents were included.

### 2.2. Collection of Compliance Data

Parental compliance was assessed as the monthly usage times and duration of the mobile app. The mobile app included 4 modules: (1) information diffusion (parents and children accessed health knowledge); (2) behavior monitoring (parents were required to record their children’s diet and exercise behaviors weekly); (3) weight management (view the recent weight status and changes and receive the individualized feedback monthly); (4) assessment and feedback (parents and children received the assessment results which were automatically generated using the mobile app weekly). Parents were encouraged to use the mobile app during the 9-month intervention; the specific content of each module and the frequency of required actions by parents can be seen in Table S1. When parents used any function of the mobile app, the background would automatically record the number of usage times and duration of a single use. We counted parents’ usage times and duration in minutes per month. In the first month, various parents started at different times during the month, so data from the first month of the intervention were excluded. As a result, app usage data during the subsequent 8-month intervention period collected from 1 October 2018 to 31 May 2019 were used for the analyses.

To assess children’s compliance, we used data recorded by parents in the “Behavior Monitoring” module of the app. To avoid increasing children’s screen time, the app was installed on the parents’ smartphones. Parents were instructed to observe and/or inquire about their child’s dietary and physical activity behaviors and then report the information accordingly. The study focused on seven key health behaviors: drinking sugary beverages, eating Western fast food, eating fried food, eating unhealthy snacks, overeating, screen time of more than 1 h, and at-home exercise. Each week, parents were asked to record their child’s behaviors. Scores were assigned based on the frequency of each behavior during the week, with 5 points awarded for meeting the recommended standard for each item, yielding a maximum total score of 35 points. For example, for drinking sugary beverages, if the child does not drink them once a week, give 5 points; if they drank them 1 or 2 times, give 4 points; if they drank them from 3 to 4 times, give 3 points; if they drank them from 5 to 6 times, give 2 points; and if they drank them more than 7 times, give 1 point. The detailed scoring methods can be seen in Table S2. According to the study protocol, parents were required to complete 7 questions weekly, for a total of 35 times. The average of each month’s behavior score was calculated as the children’s compliance for a total of 9 months. The proportion of missing values was about 10% for each month. Missing data were imputed using a two-step approach. For the first time point with missing data, the median score was used due to the non-normal distribution of the data. To account for behavioral continuity, later missing values were imputed using the most recent observed value. We compared the baseline characteristics among participants with complete data and those with missing data. No significant differences were observed among these groups (Table S3).

### 2.3. The Influencing Factors of Compliance

The baseline questionnaire of the study collected sociodemographic information including children’s age, gender, primary caregiver, parents’ education level, parental age, parental obesity indicators (body mass index [BMI], obese or overweight), whether the mother was employed, and whether the child was the only child in the family. We also included the obesity indicators of children (BMI, BMI z score, obese or overweight, waist circumference, and body fat percentage) measured at baseline.

### 2.4. Outcome Measurements

Baseline measurements was conducted in September 2018, and follow-up measurements was conducted 9 months after the baseline measurements in June 2019. We measured anthropometric indicators: height (Stadiometer, Huateng GMCS-1, Beijing, China), weight (Lever scale, Wujin RGT-140, Beijing, China), waist circumference (Tape, MyoTape, Beijing, China), hip circumference (Tape, MyoTape, Beijing, China), and body fat percentage (BF%) (Body component instrument, Tanita MC-780 MA, Tokyo, Japan) before and after the intervention. All assessments were performed by trained staff according to the standard protocol. BMI was calculated as weight in kilograms divided by the square of height in meters. The age and sex-specific BMI z score was calculated according to the child growth and development standards released by the World Health Organization [14]. Waist-to-hip ratio (WHR) was calculated by dividing waist circumference by hip circumference.

### 2.5. Statistical Analyses

#### 2.5.1. Compliance Trajectory Analysis

The group-based trajectory model (GBTM) is a type of latent class analysis that incorporates information on its dynamic nature and classifies individuals into different trajectories over time with easily interpretable graphics [17]. We used the GBTM to identify trajectory groups of parental compliance and child compliance. The model was established with month as the independent variable, usage times and duration of the mobile app, and children’s monthly behavior scores as the outcome variables. We conducted and compared models with 2–4 groups for parental compliance and 3–6 groups for children’s compliance. The final model selection was based on the following criteria: (1) The smallest group had to include at least 7% of the sample. (2) Bayesian Information Criterion (BIC): the value closest to 0 indicates the best-fitting model. (3) Each group’s average posterior probability (AvePP) had to be more than 0.7. (4) The odds of correct classification (OCC) for each group had to be more than 5.0.

#### 2.5.2. Effect of Compliance on Obesity-Related Anthropometrics

To ascertain the significance of good compliance, we did linear mixed models to analyze the association between compliance trajectory groups and outcomes related to obesity (BMI, BMI z score, waist circumference, WHR, BF%). The outcomes in the model were the changes in anthropometric measures. We adjusted for students’ age, sex, and baseline anthropometric measures, incorporating a class-level random intercept.

#### 2.5.3. Influencing Factors of Trajectories

A descriptive summary of the baseline anthropometric measures (BMI, BMI z score, waist circumference, BF%) and influencing factors (such as students’ age, gender, region, overweight and obesity, whether they are the only child, father’s education level, mother’s education level, whether the main caregiver is the mother, and whether the mother has a job, et al.) of different trajectories were analyzed. Continuous variables were described as the mean and standard deviation (SD) if they were normally distributed, and categorical variables were described as the number of cases and percentage (%). Independent-sample *t*-tests were used to assess whether the anthropometric measures and influencing factors differed by compliance trajectory group.

Logistic regression was conducted to investigate the factors associated with parental and child compliance trajectories. Parental and child compliance trajectories were examined as the dependent variable, and the characteristics of children and parents were examined as the independent variables. Univariate logistic regression models were initially conducted. Variables that showed statistical significance with outcomes in the univariate analysis were included in the multivariate logistic regression models.

The results were considered statistically significant at two-sided *p* < 0.05. Statistical analyses were carried out using Stata 17 [18] and R software (version 4.1.2; creator: John Chambers and colleagues; location: Jersey City, NJ, USA).

## 3. Results

### 3.1. Parental Compliance Trajectory

**Figure 1:**
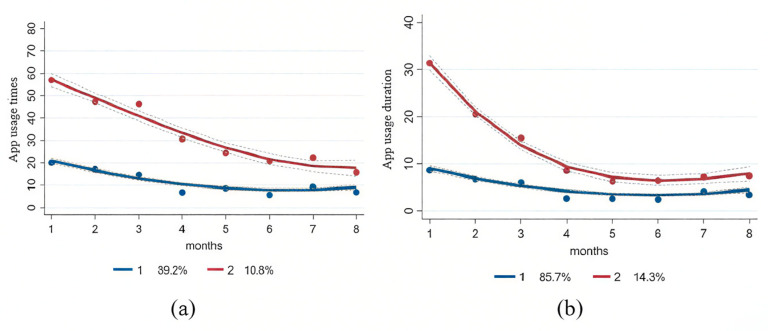
Trajectory group models with two groups for the app usage times and app duration (minutes) of parents. (**a**) Two trajectory groups for app usage times. (**b**) Two trajectory groups for app usage duration.

For parents, both app usage time and duration generated different trajectory models ranging from two to four compliance trajectory groups (Table S4). The optimal group-based trajectory model, the two-group model, was selected according to the criteria (Figure 1). In this model, parents were classified into two distinct groups based on their app usage times and duration trajectories during the period of intervention. As illustrated in Figure 1, the “relatively low” trajectory group for app usage times (*n* = 612, 89.2%) showed a decline in the mean (SD) number of clicks from 19.94 (13.09) times at the baseline month to 6.81 (6.65) times at the end of the intervention. The other group, referred to as the “relatively high” trajectory group (*n*  =  74, 10.8%), started at a mean (SD) number of 56.88 (26.63) times at the baseline month and 15.63 (10.89) times at the end of the intervention. Regarding app usage duration, both trajectory groups demonstrated declining trends, consistent with those observed for usage times. In the ‘relatively low’ group, the mean (SD) monthly duration decreased from 8.62 (5.34) minutes at baseline to 3.43 (3.57) minutes at the end of the intervention. In the “relatively high” group, the duration declined from 31.47 (14.71) minutes to 7.44 (6.94) minutes per month.

### 3.2. Children’s Compliance Trajectory

**Figure 2:**
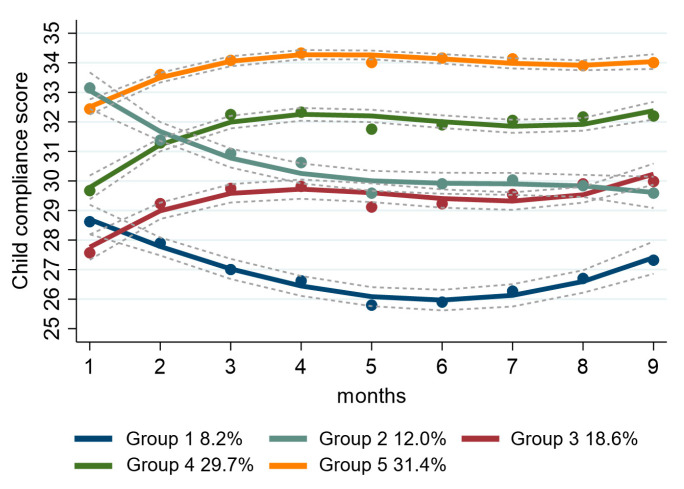
Trajectory group models with five groups for children’s compliance.

For children’s compliance, we identified five differential compliance trajectories (Figure 2, Table S5). The five compliance trajectories are as follows: Group 1 (initially low and decreasing, 8.2%), Group 2 (initially high but decreasing, 12.0%), Group 3 (initially poor but improving, 18.6%), Group 4 (moderate improvement, 29.7%), and Group 5 (high and sustained improvement, 31.4%). The description of baseline characteristics in these five groups was shown in Table S6. For better interpretation, we merged five groups into two categories: the improving compliance group (comprising groups 3, 4, and 5, accounting for 79.8%) and the decreasing compliance group (consisting of groups 1 and 2, making up 20.2%).

### 3.3. Different Compliance Trajectories and Obesity-Related Outcomes

For parental compliance trajectories, compared to the “relatively low” trajectory group, the “relatively high” trajectory group had a significant effect of intervention on waist circumference (app usage times: β = −1.17, 95% CI: −1.98, −0.36, *p* = 0.01; app duration: β = −1.03, 95% CI: −1.76, −0.30, *p* = 0.01) and WHR (app usage times: β = −0.01, 95% CI: −0.02, −0.00, *p* < 0.01; app duration: β = −0.01, 95% CI: −0.02, −0.00, *p* = 0.01) after intervention. This indicates that higher parental compliance was associated with an average reduction of approximately 1 cm in waist circumference, which may contribute to a clinically meaningful decrease in central adiposity and related metabolic risks. As for children’s compliance trajectories, compared to the decreasing compliance group, children in the improving compliance group achieved a significantly greater reduction in BF% after the intervention (β = −0.65, 95% CI: −0.04, −1.26, *p* = 0.04). This reduction in BF% suggests that improvements in child compliance over time may contribute to measurable improvements in body composition (Table 1).

### 3.4. The Influencing Factors of Parental Compliance

For app usage times, after adjusting for other influencing factors in the multivariate logistic regression, having a daughter remained significantly associated with the “relatively low” parental compliance trajectory (OR: 1.95, 95% CI: 1.17–3.31). For app usage duration, the multivariate logistic regression showed that having a highly educated father was significantly associated with the “relatively low” parental compliance trajectory (OR: 1.65, 95% CI: 1.05–2.60) (Table 2 and Table S7).

### 3.5. The Influencing Factors of Children’s Compliance

We found that children being male (OR: 1.59, 95%: 1.09, 2.33), younger maternal age (OR: 1.05, 95%: 1.01–1.10), and low parental compliance (lower app usage times) (OR: 2.29, 95%: 1.13, 5.27) were associated with the decreasing children compliance group. Children with low baseline BMIs were associated with the improving child compliance group (OR: 0.95, 95%: 0.91, 0.99). In multivariate logistic regression, after adjusting for other factors, younger maternal age (OR: 1.05, 95%: 1.01, 1.10) and low parental compliance (OR: 2.36, 95%: 1.16, 5.47) remained associated with the group exhibiting decreasing children compliance (Table 3 and Table S8).

## 4. Discussion

To the best of our knowledge, this is the first study to employ longitudinal data to identify distinct compliance trajectories and examine the influencing factors underlying changes in compliance over the course of a childhood obesity intervention. We found that the compliance of parents had a declining trend, while the majority of children exhibited improving compliance. After identifying trajectory groups through a GBTM analysis, we observed a correlation between the poor compliance group and reduced child obesity-related outcomes. The child being female and highly educated fathers were linked to “relatively low” parental compliance. Additionally, younger maternal age and poor parental compliance were associated with decreasing child compliance.

We observed that app usage compliance gradually deteriorated during the intervention. A systematic review of parental-focused obesity mHealth interventions concluded that the dropout rates in the included studies ranged from 12% to 29% [19]. The loss of engagement and declined compliance were not only one of the challenges of the application in obesity interventions [20], but also a problem that exists in most intervention studies. Koorts et al. [21] found that, as the obesity intervention progressed, there was a decline in the delivery of key messages and other curriculum-related components over time. Commonly, interventions do not continue as originally implemented and show unintentional “slippage” [22]. This study provides novel evidence of a declining trend in compliance by analyzing the trajectories of compliance changes. Intervention compliance should be examined as a dynamic phenomenon, necessitating monitoring and timely reminders throughout the study.

Methodological alternatives could add some insights into the study of compliance in the field of childhood obesity. Despite the extensive use of GBTM in the fields of medical research and medication adherence [23,24], no application has been seen in the study of compliance with childhood obesity interventions. Parental compliance data had a time-based dimension with individual variabilities and a population mean, and children’s dieting and exercise behavior may improve to varying degrees depending on individual variability, which provided the empirical foundation for the analysis of developmental trajectories [25]. We should note that conventional methods, such as calculating an average for overall compliance, may lead to the mixing and misclassification of groups with similar average levels. This makes it challenging to identify internal features accurately. From this perspective, employing the GBTM trajectory method enables the distinction of groups with similar average levels, offering more insights than a single average value. Researchers can thereby explore more detailed information about the various change patterns.

Low parent compliance and decreasing child compliance were associated with reduced anthropometric outcomes. Other studies also provided evidence that better compliance was associated with better intervention outcomes [26,27,28], underscoring the importance of increased attention to compliance in obesity intervention studies. In addition, our research found the positive effect of parental compliance on child compliance. As the previous literature showed, parents’ motivation and behavior were the key influencers on the development of their children’s healthy behaviors [29]. Support from family members was the determinant of adherence to the intervention for adolescents with obesity [30]. A meta-synthesis showed that, compared to minimal or no family involvement, a high level of family engagement was associated with greater reductions in obesity [31]. These findings emphasize the importance of parent engagement in childhood obesity prevention interventions.

The results of our study identify important population characteristics affecting parents’ and children’s compliance. Clarifying these characteristics associated with high intervention compliance would contribute valuable insights on who to target for promoting intervention compliance [32]. Our findings reveal that parental compliance differed by child sex, and the parents of boys showed better compliance. Previous obesity intervention in school was particularly effective in girls [33,34,35], which may be attributed to the fact that girls generally exhibited higher compliance and parents typically did not need to provide additional support for them [28]. Another weight loss intervention program also indicated that boys were more likely to drop out than girls and that gender could predict treatment attrition [14]. The parents may require more effort to assist boys in maintaining good compliance and improving healthy behaviors. Apart from child sex, we found that a father’s high education level was associated with low parental compliance. Although the correlation between maternal education level and compliance was not significant, there was still a trend in our study, with a higher proportion of mothers with high education levels in the low compliance group. High paternal education levels may be associated with more demanding occupations that have long working hours [36], resulting in less time and energy for child-rearing responsibilities, a primary barrier in family-based obesity interventions [37]. However, in most families, fathers often play a relatively limited role in daily caregiving compared to mothers, especially regarding health-related routines [38]. Considering the important potential health effects of fathers on their children [39], future interventions should consider strategies to engage fathers more actively, addressing both time constraints and culturally rooted role expectations.

As for children’s compliance, we found that older maternal age was the factor associated with higher child compliance. Existing evidence suggests a positive correlation between maternal age and favorable parental behaviors [40,41]. This correlation contributes to a reduction in social and emotional difficulties, as well as an improvement in social skill scores, including self-control [42], providing a plausible explanation for the observed association between maternal age and child compliance.

The strengths of this study included several aspects. We utilized the mobile app to obtain continuous and accurate records of parents’ app usage times and duration, objectively measuring parental compliance. Additionally, we simultaneously took into account the compliance of the children themselves, using the health behavior scores recorded multiple times in the mobile app. Furthermore, to make full use of the monitoring data, we employed GBTM trajectory analysis. This approach had advantages over other traditional methods of compliance assessment, since considering the dynamic nature of compliance. The findings of this study also had implications for future childhood intervention studies. To promote good compliance among intervention subjects, besides enhancing the practical feasibility of the research protocol, researchers could identify populations with potentially low compliance based on the relevant factors. For instance, increased supervision may be necessary for parents of female children and those with higher levels of education.

This study has several limitations. First, in this study, the influencing factors are limited. Additional characteristics, such as parents’ health literacy and income level, as well as the child’s academic levels (serving as an indicator of their capacity to comprehend health knowledge), might influence compliance. Future intervention studies should collect the data of those potential factors. Second, our study did not perform qualitative interviews to elucidate how these characteristics impact compliance. In future research, integrating mixed methods approaches—such as combining quantitative analysis with qualitative interviews—could provide a deeper understanding of the “why” behind compliance patterns and help uncover the contextual or psychosocial factors affecting participation. Third, the children’s compliance data were collected from the parents’ perspective, which may have influenced the accuracy of the reported compliance and behaviors. However, since the data were based on parents’ direct observations and inquiries into their children’s daily activities, they may still offer a relatively reliable representation of the children’s behaviors.

## 5. Conclusions

This study identified distinct parental and child compliance trajectories over time in an mHealth-assisted childhood obesity intervention. Both student and parental compliance were shown to influence the effectiveness of childhood obesity interventions, highlighting the need to prioritize the assessment and promotion of compliance in such interventions. Child sex, paternal educational level, and maternal age were identified as significant factors associated with compliance, while the level of family involvement was found to play a pivotal role in fostering healthy behaviors in children. These findings suggest that future intervention strategies should place greater emphasis on engaging families and providing targeted supervision and support for populations at risk of lower compliance in order to enhance intervention outcomes.

## Figures and Tables

**Table 1:** Different trajectory and obesity-related outcomes.

	Parental Compliance App Usage Times		Parental Compliance App Usage Duration		Child Compliance	
	“Relatively High” vs. “Relatively Low”	*p*	“Relatively High” vs. “Relatively Low”	*p*	Improving Compliance Group vs. Decreasing Compliance Group	*p*
Change in BMI (mean (SD))	−0.14 (−0.35, 0.08)	0.22	−0.11 (−0.30, 0.09)	0.28	−0.11 (−0.28, 0.05)	0.17
Change in BMI z score (mean (SD))	−0.04 (−0.13, 0.05)	0.36	−0.03 (−0.11, 0.05)	0.43	−0.03 (−0.10, 0.04)	0.36
Change in waist circumference (mean (SD))	**−1.17 (−1.98, −0.36)**	**0.01**	**−1.03 (−1.76, −0.30)**	**0.01**	−0.19 (−0.81, 0.43)	0.54
Change in WHR (mean (SD))	**−0.01 (−0.02, −0.00)**	**0.01**	**−0.01 (−0.02, −0.00)**	**0.01**	0.00 (−0.01, 0.01)	0.88
Change in BF% (mean (SD))	−0.30 (−1.11, 0.51)	0.44	−0.15 (−0.87, 0.57)	0.69	**−0.65 (−0.04, −1.26)**	**0.04**

“Relatively low” group of app usage times and duration in parents; the decreasing compliance group in children was the reference group. Abbreviations: BMI, body mass index; WHR, waist-to-hip ratio; BF%, body fat percentage. The significant results were bolded.

**Table 2:** Univariate and multivariate logistic regression analysis of factors influencing app usage compliance (“relatively high” compliance group as reference).

Characteristics	Level	App Usage Times		OR (95% CI) ^a^	OR (95% CI) ^b^	App Usage Duration		OR (95% CI) ^a^	OR (95% CI) ^b^
		Low (*n* = 610)	High (*n* = 74)			Low (*n* = 586)	High (*n* = 98)		
Students									
Gender (%)	Boy	297 (48.7)	46 (62.2)	1.72 (1.06, 2.86)	1.95 (1.17, 3.31)	-	-	-	-
	Girl	313 (51.3)	28 (37.8)			-	-		-
Single child (%) ^#^	Yes	373 (61.8)	35 (47.3)	1.80 (1.11, 2.94)	1.65 (0.96, 2.86)	-	-	-	-
	No	231 (38.2)	39 (52.7)			-	-		-
Age (Mean ± SD))		-	-	-		9.61 (0.34)	9.69 (0.37)	1.97 (1.07, 3.66)	1.70 (0.89, 3.25)
Parents									
Mother age (year, mean ± SD)		-	-	-	-	37.73 (4.20)	38.69 (4.43)	1.05 (1.01, 1.11)	1.03 (0.98, 1.09)
Father’s education level (%) *	High	-	-	--	--	321 (56.4)	41 (42.7)	1.74 (1.13, 2.70)	1.65 (1.05, 2.60)
	Low	-	-			248 (43.6)	55 (57.3)		
Mother’s education level (%) *	High	362 (61.1)	35 (48.6)	1.67 (1.02, 2.73)	1.21 (0.70, 2.09)	-	-	-	-
	Low	230 (38.9)	37 (51.4)			-	-		-
Mother with the job (%) ^#^	Yes	-	-	-	-	483 (85.5)	72 (76.6)	1.79 (1.03, 3.01)	1.51 (0.85, 2.59)
	No	-	-			82 (14.5)	22 (23.4)		

Only variables that were statistically significant for the outcome in univariate analyses are shown. ^a^: The results of the univariate logistic regression analysis. ^b^: The results of the multivariate logistic regression analysis. *: “high” means a college degree or above and “low” means a low college degree. ^#^: There is a missing value, so the synthesis is not 684, and the missing value appears as random missing.

**Table 3:** Univariate and multivariate logistic regression analysis of factors influencing child compliance (improving compliance group as reference).

Characteristics	Level	Improving Compliance Group	Decreasing Compliance Group	OR (95% CI) ^a^	OR (95% CI) ^b^
		*n* = 542	*n* = 140		
Students					
Gender (%)	Boy	259 (47.8)	83 (59.3)	1.59 (1.09, 2.33)	1.47 (0.99, 2.19)
	Girl	283 (52.2)	57 (40.7)		
BMI (Mean ± SD)		18.40 (3.59)	19.11 (4.03)	0.95 (0.91, 0.99)	0.96 (0.91, 1.01)
Parents					
Mother age (year, mean ± SD)		38.04 (4.16)	37.24 (4.49)	1.05 (1.01, 1.10)	1.05 (1.01, 1.10)
Parental compliance (app usage times)	Low	476 (87.8)	132 (94.3)	2.29 (1.13, 5.27)	2.36 (1.16, 5.47)
	High	66 (12.2)	8 (5.7)		

^a^ The results of the univariate logistic regression analysis. ^b^ The results of the multivariate logistic regression analysis.

## Data Availability

Data of this study would be available by contacting the corresponding authors and could be provided with the consent of the Ethics Committee.

## Supplementary Materials

The following supporting information can be downloaded at: https://www.mdpi.com/article/10.3390/nu17101701/s1, Table S1: Four modules, detailed contents, and the frequency of completion; Table S2: Score corresponding to the frequency of diet and exercise behaviors within a week; Table S3: Comparison of the population characteristics with and without missing data; Table S4: Model fit statistics (parents); Table S5: Model fit statistics (children); Table S6: Baseline characteristics of children’s compliance trajectory groups; Table S7: Baseline characteristics of parental compliance trajectory groups and Univariate Logistic Regression Analysis (“relatively high” Compliance Group as Reference); Table S8: Baseline characteristics of child compliance trajectory groups and Univariate Logistic Regression Analysis (Improving Compliance Group as Reference).

## Abbreviations

The following abbreviations are used in this manuscript:

GBTM	Group-based trajectory modeling
DECIDE-Children	Diet, ExerCIse, and CarDiovascular hEalth Children
BMI	Body Mass Index
BF%	Body fat percentage
WHR	Waist-to-hip ratio
AvePP	Average posterior probability
OCC	Odds of correct classification
SD	Standard deviation
