# Supplementary material for: Identifying Longitudinal Compliance Patterns and Determinants in a Multifaceted Childhood Obesity Intervention Using Group-Based Trajectory Modeling
Source: Nutrients. 2025 May 16;17(10):1701. doi: 10.3390/nu17101701 (PMC12114602; doi:10.3390/nu17101701)
Supplement: Supplementary file 1 [file nutrients-17-01701-s001.zip › nutrients-3613030-supplementary.pdf]

Supplemental materials

Table S1 Four modules, detailed contents, and the frequency of completion.

Modules	Contents	Frequency
Information diffusion	Regularly sending knowledge points related to obesity prevention to parents and students.	At least 15 times throughout the intervention period.
Behavior monitoring	Parents recorded the students' dieting and physical activity behaviors and completed the behavior questionnaire every week.	35 times.
Weight management	Parents were required to view the recent weight status and changes of their children, and learn the individualized feedback monthly.	9 times.
Assessment and feedback	According to students' weight and behavior monitoring information, the mobile App would automatically generate the comprehensive assessment results, which were returned weekly to parents and students. The content would include the results of weight monitoring, weight change assessment, diet, and exercise behavior assessment. Parents and students could access the assessment results through the mobile App.	At least 35 times throughout the intervention period.

Table S2 Score corresponding to the frequency of diet and exercise behaviors within a week

Part	5 points	4 points	3 points	2 points	1 point
Drinking sugary beverages	0	1~2 times	3~4 times	5~6 times	More than 7 times

Eating western fast food	0	1~2 times	3~4 times	5~6 times	More than 7 times
Eating fried food	0	1~2 times	3~4 times	5~6 times	More than 7 times
Eating unhealthy snacks	0	1~2 times	3~4 times	5~6 times	More than 7 times
Overeating	0	1~2 times	3~4 times	5~6 times	More than 7 times
Screen time of more than 1 hour	0	1~2 days	3~4 days	5~6 days	More than 7 days
At-home exercise	Following the protocol	More than half an hour a day of other forms of exercise.	-	Less than half an hour a day of other forms of exercise	No exercise

Table S3 Comparison of the population characteristics with different numbers of missing data

Characteristics	level	0	1	2	⩾3	P
		n=95	n=249	n=116	n=224	
**Students**						
Gender (%)	Boy	46 (48.4)	118 (47.4)	62 (53.4)	117 (52.2)	0.62
	Girl	49 (51.6)	131 (52.6)	54 (46.6)	107 (47.8)	
Nation (%)#	Han	85 (90.3)	226 (90.8)	107 (93.0)	208 (92.9)	0.76
	Other	10 (9.7)	23 (9.2)	8 (7.0)	16 (7.1)	
Single child (%)#	Yes	53 (55.9)	148 (59.9)	68 (59.1)	139 (62.9)	0.70
	No	42 (44.1)	99 (40.1)	47 (40.9)	82 (37.1)	
Age (Mean±SD))		9.67 (0.33)	9.62 (0.34)	9.65 (0.38)	9.60 (0.34)	0.31
BMI (Mean±SD)		17.90 (3.14)	18.54 (3.85)	18.64 (3.51)	18.76 (3.82)	0.30
BMI Z score (Mean±SD)		0.47 (1.31)	0.67 (1.47)	0.78 (1.32)	0.79 (1.49)	0.31
Obese or overweight (%)	Yes	29 (31.2)	95 (38.2)	47 (40.5)	94 (42.0)	0.33
	No	64 (68.8)	154 (61.8)	69 (59.5)	130 (58.0)	
WC (cm, mean±SD)		63.55 (8.68)	65.11 (10.56)	65.39 (10.04)	65.74 (10.51)	0.38
WHR (Mean±SD)		0.85 (0.05)	0.86 (0.06)	0.86 (0.06)	0.86 (0.06)	0.84
BF% (Mean±SD)		19.07 (8.75)	21.10 (11.06)	20.68 (9.85)	20.98 (10.63)	0.43

**Parents**						
Mother age (year, mean±SD)		38.16 (4.52)	38.32 (4.16)	37.41 (3.65)	37.51 (4.46)	0.11
Father age (year, mean±SD)		40.05 (5.07)	40.39 (4.63)	39.37 (4.53)	39.52 (4.82)	0.14
Father BMI (Mean±SD)		24.15 (4.71)	24.26 (4.12)	24.14 (4.18)	24.21 (4.35)	0.99
Mother BMI (Mean±SD)		21.45 (4.84)	21.87 (3.77)	21.82 (3.87)	22.22 (4.01)	0.47
Fathers are overweight or obese (%)#	Yes	47 (56.6)	137 (58.1)	64 (58.2)	115 (56.1)	0.97
	No	36 (43.4)	99 (41.9)	46 (41.8)	90 (43.9)	
Mothers are overweight or obese (%)#	Yes	27 (29.7)	50 (20.8)	24 (21.4)	60 (28.3)	0.15
	No	64 (70.3)	190 (79.2)	88 (78.6)	152 (71.7)	
Primary caregiver (%)	Mother	76 (81.7)	186 (74.7)	90 (77.6)	155 (69.2)	0.09
	Father	17 (18.3)	63 (25.3)	26 (22.4)	69 (30.8)	
Father's education level (%)#	High [a]	44 (47.8)	140 (58.1)	59 (51.8)	118 (54.6)	0.35
	Low [a]	48 (52.2)	101 (41.9)	55 (48.2)	98 (45.4)	
Mother's education level (%)#	High [a]	48 (52.7)	155 (64.0)	68 (59.6)	125 (58.1)	0.27

	Low [a]	43 (47.3)	87 (36.0)	46 (40.4)	90 (41.9)	
Mother with the job (%)#	Yes	75 (83.3)	198 (82.8)	94 (83.2)	186 (86.5)	0.72
	No	15 (16.7)	41 (17.2)	19 (16.8)	29 (13.5)	

Abbreviation: BMI, body mass index; WC, waist circumference; BF%, body fat percentage.

a "high" means a college degree or above, and "low" means a low college degree.

Table S4 Model fit statistics (parents)

Groups in trajectory modeling	BIC (N=5472)	BIC (N=684)	AIC	>7% per group
**App usage times**				
2	-20048.64	-20038.25	-20015.53	Yes
3	-19805.43	-19789.84	-19755.77	No
4	-19690.09	-19669.30	-19623.88	No
**App Usage Time**				
**Groups in trajectory modeling**	**BIC (N=5472)**	**BIC (N=684)**	**AIC**	**>7% per group**
2	-16607.16	-16596.76	-16574.05	Yes
3	-16421.07	-16405.47	-16371.40	No
4	-16414.84	-16394.04	-16348.62	No

Abbreviation: BIC, Bayesian Information Criterion; AIC, Akaike Information Criterion

Table S5 Model fit statistics (children)

Groups in trajectory modeling	BIC (N=6156)	BIC (N=684)	AIC	>7% per group
3	-13586.96	-13570.48	-13536.54	Yes
4	-13466.12	-13444.15	-13398.90	Yes
5	-13377.20	-13349.73	-13293.17	Yes
6	-13333.99	-13301.03	-13233.15	No

Table S6 Baseline characteristics of children's compliance trajectory groups

Characteristics	level	Group 1	Group 2	Group 3	Group 4	Group 5	P
		n=59	n=83	n=123	n=205	n=214	
**Students**							
Gender (%)	Boy	38 (64.4)	47 (56.6)	66 (53.7)	99 (48.3)	94 (43.9)	0.04
	Girl	21 (35.6)	36 (43.4)	57 (46.3)	106 (51.7)	120 (56.1)	
Nation (%)#	Han	52 (88.1)	74 (90.2)	111 (90.2)	186 (90.7)	202 (94.4)	0.56
	Other	7 (11.9)	8 (9.8)	12 (9.8)	19 (9.3)	12 (5.6)	
Single child (%)#	Yes	39 (67.2)	50 (61.7)	85 (69.7)	118 (57.8)	115 (54.5)	0.06
	No	19 (32.8)	31 (38.3)	37 (30.3)	86 (42.2)	96 (45.5)	
Age (Mean±SD))		9.62 (0.32)	9.64 (0.37)	9.61 (0.33)	9.62 (0.37)	9.63 (0.34)	0.94
BMI (Mean±SD)		19.35 (3.90)	18.94 (4.14)	19.49 (4.29)	18.42 (3.48)	17.74 (3.09)	0.00
BMI Z score (Mean±SD)		1.01 (1.49)	0.80 (1.58)	1.01 (1.55)	0.68 (1.37)	0.41 (1.30)	0.00
Obese or overweight (%)	Yes	29 (50.0)	33 (40.2)	60 (48.8)	81 (39.5)	62 (29.0)	0.00
	No	29 (50.0)	49 (59.8)	63 (51.2)	124 (60.5)	152 (71.0)	
WC (cm, mean±SD)		67.20 (10.69)	66.12 (11.63)	67.58 (11.35)	64.94 (9.94)	63.03 (8.61)	0.00

WHR (Mean±SD)		0.87 (0.06)	0.86 (0.06)	0.86 (0.06)	0.86 (0.06)	0.85 (0.05)	0.14
BF% (Mean±SD)		22.09 (11.31)	21.35 (11.02)	23.33 (11.48)	20.27 (9.95)	19.01 (9.45)	0.00
**Parents**							
Mother age (year, mean±SD)		36.92 (4.92)	37.48 (4.16)	37.70 (3.58)	37.68 (4.16)	38.60 (4.43)	0.03
Father age (year, mean±SD)		39.83 (5.20)	39.21 (4.91)	39.35 (3.86)	39.61 (4.96)	40.75 (4.74)	0.03
Father BMI (Mean±SD)		25.01 (4.47)	23.66 (5.01)	24.53 (4.55)	24.25 (4.03)	23.97 (3.98)	0.35
Mother BMI (Mean±SD)		22.77 (4.24)	21.84 (4.68)	21.80 (4.42)	21.64 (3.48)	22.05 (3.94)	0.43
Fathers are overweight or obese (%)[#]	Yes	31 (56.4)	40 (53.3)	67 (57.3)	110 (57.9)	115 (58.4)	0.96
	No	24 (43.6)	35 (46.7)	50 (42.7)	80 (42.1)	82 (41.6)	
Mothers are overweight or obese (%)[#]	Yes	17 (29.8)	22 (27.8)	31 (25.8)	38 (19.5)	53 (26.0)	0.36
	No	40 (70.2)	57 (72.2)	89 (74.2)	157 (80.5)	151 (74.0)	
Primary caregiver (%)	Mother	38 (65.5)	65 (78.3)	95 (77.2)	155 (75.6)	154 (72.0)	0.31
	Father	21 (34.5)	18 (21.7)	28 (22.8)	50 (24.4)	60 (28.0)	
Father's education level (%)[#]	High [a]	29 (49.2)	51 (63.8)	73 (59.3)	114 (57.3)	94 (46.1)	0.03
	Low [a]	30 (50.8)	29 (36.2)	50 (40.7)	85 (42.7)	110 (53.9)	
Mother's education level (%)[#]	High [a]	33 (55.9)	55 (68.8)	86 (71.1)	116 (58.3)	107 (52.2)	0.00
	Low [a]	26 (44.1)	25 (31.2)	35 (28.9)	83 (41.7)	98 (47.8)	

Mother with the job (%)#	Yes	53 (89.8)	69 (85.2)	104 (86.7)	161 (82.6)	166 (81.4)	0.32
	No	6 (10.2)	12 (14.8)	16 (13.3)	34 (17.4)	38 (18.6)	
Parental compliance (App usage time)	Low	39 (67.2)	43 (52.4)	61 (49.6)	85 (41.5)	112 (52.3)	0.01
	High	19 (32.8)	39 (47.6)	62 (50.4)	120 (58.5)	102 (47.7)	
Parental compliance (App usage times)	Low	46 (79.3)	52 (63.4)	61 (49.6)	96 (46.8)	85 (39.7)	0.00
	High	12 (20.7)	30 (36.6)	62 (50.4)	109 (53.2)	129 (60.3)	

Abbreviation: BMI, body mass index; WC, waist circumference; BF%, body fat percentage.

[a] "high" means a college degree or above, and "low" means a low college degree.

#: There is a missing value, so the synthesis is not 684, and the missing appears as random missing.

Table S7 Baseline characteristics by parental compliance trajectory groups and Univariate Logistic Regression Analysis("relatively low" Compliance Group as Reference)

Characteristics	level	App usage times		OR(95% CI)	*P*	App duration		OR(95% CI)	*P*
		Low(n=610)	High(n=74)			Low(n=586)	High(n=98)		
**Students**									
Gender (%)	Boy	297 (48.7)	46 (62.2)	0.58	0.04	289 (49.3)	54 (55.1)	0.79	0.34

	Girl	313 (51.3)	28 (37.8)	(0.35, 0.94)		297 (50.7)	44 (44.9)	(0.51, 1.22)	
Nation (%)#	Han	557 (91.5)	70 (94.6)	0.61	0.48	533 (91.1)	94 (95.9)	0.43	0.16
	Other	52 (8.5)	4 (5.4)	(0.18, 1.55)		52 (8.9)	4 (4.1)	(0.13, 1.09)	
Single child (%)#	Yes	373 (61.8)	35 (47.3)	1.80	0.02	351 (60.5)	57 (58.2)	1.1	0.74
	No	231 (38.2)	39 (52.7)	(1.11, 2.94)		229 (39.5)	41 (41.8)	(0.71, 1.7)	
Age (Mean±SD))		9.62 (0.35)	9.65 (0.36)	1.31 (0.65, 2.61)	0.46	9.61 (0.34)	9.69 (0.37)	1.97 (1.07, 3.66)	0.03
Behavior scores(%)	Low	322 (53.0)	19 (25.7)	3.26 (1.92, 5.76)	0.01	301 (51.5)	40 (40.8)	1.54 (1.00, 2.39)	0. 05
	High	286 (47.0)	55 (74.3)			283 (48.5)	58 (59.2)		
BMI (Mean±SD)		18.54 (3.71)	18.54 (3.61)	1.00 (0.93, 1.07)	0.99	18.49 (3.66)	18.82 (3.91)	1.02 (0.97, 1.08)	0.42
BMI z score (Mean±SD)		0.70 (1.43)	0.71 (1.49)	1.00 (0.85, 1.19)	0.95	0.68 (1.44)	0.78 (1.43)	1.04 (0.9, 1.21)	0.56
Obese or overweight (%)	Yes	233 (38.2)	33 (44.6)	0.77	0.35	226 (38.6)	40 (40.8)	0.91	0.76
	No	377 (61.8)	41 (55.4)	(0.47, 1.25)		360 (61.4)	58 (59.2)	(0.59, 1.41)	
waist circumference (cm, mean ±SD)		65.07 (10.26)	65.84 (10.04)	1.01 (0.98, 1.03)	0.54	64.99 (10.21)	66.13 (10.33)	1.01 (0.99, 1.03)	0.31

BF% (Mean±SD)		20.73 (10.41)	20.59 (10.62)	1.00 (0.98, 1.02)	0.92	20.61 (10.32)	21.34 (11.07)	1.01 (0.99, 1.03)	0.52
**Parents**									
Mother age (year, mean±SD)		37.86 (4.25)	37.90 (4.17)	1.00 (0.94, 1.06)	0.94	37.73 (4.20)	38.69 (4.43)	1.05 (1.01, 1.11)	0.04
Father age (year, mean±SD)		39.89 (4.75)	39.82 (4.72)	1.00 (0.95, 1.05)	0.90	39.74 (4.69)	40.74 (4.98)	1.04 (1.00, 1.09)	0.06
Father BMI (Mean±SD)		24.29 (4.34)	23.43 (3.82)	0.96 (0.91, 1.01)	0.11	24.32 (4.30)	23.46 (4.21)	0.96 (0.91, 1)	0.08
Mother BMI (Mean±SD)		21.95 (4.08)	21.55 (3.69)	0.98 (0.92, 1.04)	0.42	21.94 (3.96)	21.74 (4.51)	0.99 (0.94, 1.04)	0.66
Fathers are overweight or obese (%)#	Yes	331 (58.5)	33 (47.1)	1.58 (0.96, 2.61)	0.09	315 (57.9)	49 (53.3)	1.21 (0.77, 1.88)	0.47
	No	235 (41.5)	37 (52.9)			229 (42.1)	43 (46.7)		
Mothers are overweight or obese (%)#	Yes	150 (25.6)	12 (16.7)	1.72 (0.93, 3.43)	0.13	140 (24.9)	22 (23.4)	1.08 (0.65, 1.84)	0.86
	No	435 (74.4)	60 (83.3)			423 (75.1)	72 (76.6)		

Primary caregiver (%)	Mother	448 (73.4)	60 (81.1)	0.65	0.20	435 (74.2)	73 (74.5)	0.99	1.00
	Father	162 (26.6)	14 (18.9)	(0.34, 1.16)		151 (25.8)	25 (25.5)	(0.6, 1.6)	
Father's education level (%)#	High [a]	330 (55.6)	32 (44.4)	1.57	0.09	321 (56.4)	41 (42.7)	1.74	0.02
	Low [a]	263 (44.4)	40 (55.6)	(0.96, 2.58)		248 (43.6)	55 (57.3)	(1.13, 2.70)	
Mother's education level (%)#	High [a]	362 (61.1)	35 (48.6)	1.67	0.04	348 (61.1)	49 (52.1)	1.44	0.13
	Low [a]	230 (38.9)	37 (51.4)	(1.02, 2.73)		222 (38.9)	45 (47.9)	(0.93, 2.24)	
Mother with the job (%)#	Yes	496 (84.5)	59 (81.9)	1.20	0.70	483 (85.5)	72 (76.6)	1.79	0.04
	No	91 (15.5)	13 (18.1)	(0.61, 2.21)		82 (14.5)	22 (23.4)	(1.03, 3.01)	

Abbreviation: BMI, body mass index; waist circumference, waist circumference; BF%, body fat percentage.

[a] "high" means a college degree or above, and "low" means a low college degree.

#: There is a missing value, so the synthesis is not 684, and the missing appears as random missing.

Table S8 Baseline characteristics by child compliance trajectory groups and Univariate Logistic Regression Analysis(Decreasing Compliance Group as Reference)

Characteristics	level	Good compliance group	Poor compliance group	OR(95% CI)	*P*
		N=542	N=140		
**Students**					
Gender (%)	Boy	259 (47.8)	83 (59.3)	1.59 (1.09 , 2.33)	0.02
	Girl	283 (52.2)	57 (40.7)		
Nation (%)[#]	Han	499 (92.1)	126 (90.6)	0.84 (0.45 , 1.66)	0.59
	Other	43 (7.9)	13 (9.4)		
Single child (%)[#]	Yes	318 (59.2)	89 (64.0)	1.23 (0.84 , 1.81)	0.30
	No	219 (40.8)	50 (36.0)		
Age (Mean±SD))		9.62 (0.35)	9.63 (0.35)	0.91 (0.53 , 1.56)	0.73
BMI (Mean±SD)		18.40 (3.59)	19.11 (4.03)	0.95 (0.91 , 0.99)	0.04
BMI z score (Mean±SD)		0.65 (1.40)	0.89 (1.54)	0.89 (0.78 , 1.01)	0.08
Obese or overweight (%)	Yes	203 (37.5)	62 (44.3)	1.33 (0.91 , 1.93)	0.14
	No	339 (62.5)	78 (55.7)		

waist circumference (cm, mean ±SD)		64.78 (9.92)	66.57 (11.22)	0.98 (0.97 , 1.00)	0.07
WHR (Mean±SD)		0.86 (0.06)	0.86 (0.06)	0.21 (0.01 , 5.42)	0.34
BF% (Mean±SD)		20.47 (10.24)	21.66 (11.10)	0.99 (0.97 , 1.01)	0.23
**Parents**					
Mother age (year, mean±SD)		38.04 (4.16)	37.24 (4.49)	1.05 (1.01 , 1.10)	0.04
Father age (year, mean±SD)		39.99 (4.67)	39.47 (5.02)	1.02 (0.98 , 1.07)	0.26
Father BMI (Mean±SD)		24.21 (4.13)	24.23 (4.81)	1.00 (0.95 , 1.04)	0.95
Mother BMI (Mean±SD)		21.84 (3.89)	22.23 (4.51)	0.98 (0.93 , 1.02)	0.31
Fathers are overweight or obese (%)[#]	Yes	292 (57.9)	71 (54.6)	0.87 (0.59 , 1.29)	0.50
	No	212 (42.1)	59 (45.4)		
Mothers are overweight or obese (%)[#]	Yes	122 (23.5)	39 (28.7)	1.31 (0.85 , 1.99)	0.21
	No	397 (76.5)	97 (71.3)		
Primary caregiver (%)	Mother	404 (74.5)	103 (73.6)	0.95 (0.63 , 1.46)	0.82
	Father	138 (25.5)	37 (26.4)		

Father's education level (%)#	High [a]	282 (53.6)	79 (57.7)	1.18 (0.81 , 1.73)	0.40
	Low [a]	244 (46.4)	58 (42.3)		
Mother's education level (%)#	High [a]	309 (58.9)	87 (63.5)	1.22 (0.83 , 1.80)	0.32
	Low [a]	216 (41.1)	50 (36.5)		
Mother with the job (%)#	Yes	431 (83.0)	122 (88.4)	1.56 (0.90 , 2.84)	0.13
	No	88 (17.0)	16 (11.6)		
Parental compliance (App usage time)	Low	460 (84.9)	124 (88.6)	1.38 (0.80 , 2.52)	0.27
	High	82 (15.1)	16 (11.4)		
Parental compliance (App usage times)	Low	476 (87.8)	132 (94.3)	2.29 (1.13 , 5.27)	0.03
	High	66 (12.2)	8 (5.7)		
